# Novel Features of a PIWI-Like Protein Homolog in the Parasitic Protozoan *Leishmania*

**DOI:** 10.1371/journal.pone.0052612

**Published:** 2012-12-21

**Authors:** Prasad K. Padmanabhan, Carole Dumas, Mukesh Samant, Annie Rochette, Martin J. Simard, Barbara Papadopoulou

**Affiliations:** 1 Research Centre in Infectious Diseases, CHUL Research Centre (CHUQ) and Department of Microbiology and Immunology, Faculty of Medicine, Laval University, Quebec, Canada; 2 INRS-Institut Armand-Frappier, Laval, Canada; 3 Laval University Cancer Research Centre, Hôtel-Dieu de Québec (CHUQ), Quebec, Canada; University of Lausanne, Switzerland

## Abstract

In contrast to nearly all eukaryotes, the Old World *Leishmania* species *L. infantum* and *L. major* lack the bona fide RNAi machinery genes. Interestingly, both *Leishmania* genomes code for an atypical Argonaute-like protein that possesses a PIWI domain but lacks the PAZ domain found in Argonautes from RNAi proficient organisms. Using sub-cellular fractionation and confocal fluorescence microscopy, we show that unlike other eukaryotes, the PIWI-like protein is mainly localized in the single mitochondrion in *Leishmania*. To predict PIWI function, we generated a knockout mutant for the *PIWI* gene in both *L. infantum* (*Lin*) and *L. major* species by double-targeted gene replacement. Depletion of PIWI has no effect on the viability of insect promastigote forms but leads to an important growth defect of the mammalian amastigote lifestage *in vitro* and significantly delays disease pathology in mice, consistent with a higher expression of the *PIWI* transcript in amastigotes. Moreover, amastigotes lacking PIWI display a higher sensitivity to apoptosis inducing agents than wild type parasites, suggesting that PIWI may be a sensor for apoptotic stimuli. Furthermore, a whole-genome DNA microarray analysis revealed that loss of *Lin*PIWI in *Leishmania* amastigotes affects mostly the expression of specific subsets of developmentally regulated genes. Several transcripts encoding surface and membrane-bound proteins were found downregulated in the *Lin*PIWI*^(−/−)^* mutant whereas all histone transcripts were upregulated in the null mutant, supporting the possibility that PIWI plays a direct or indirect role in the stability of these transcripts. Although our data suggest that PIWI is not involved in the biogenesis or the stability of small noncoding RNAs, additional studies are required to gain further insights into the role of this protein on RNA regulation and amastigote development in *Leishmania*.

## Introduction

The Argonaute (AGO) proteins represent a large family of proteins that bind to small RNAs like microRNAs (miRNAs), short interfering RNAs (siRNAs) and PIWI-interacting RNAs (piRNAs) [1]. They are central to many transcriptional and posttranscriptional gene silencing pathways [2] as well as involved in transposon control, which otherwise threaten the integrity of the genome [3]. Argonaute proteins are evolutionarily conserved and can be phylogenetically classified into three paralogous groups: the AGO subfamily proteins which are similar to *Arabidopsis thaliana* AGO1, the PIWI subfamily which is closely related to *Drosophila melanogaster* PIWI and the worm-specific WAGO subfamily which is related to the *C. elegans* specific group 3 Argonaute proteins [4]. The AGO-clade proteins have been found in nearly all eukaryotes, while the PIWI-clade proteins are restricted to animals and ciliates, organisms which undergo sexual reproduction [5]. Many prokaryotes and archaea also encode homologs of Argonaute proteins but their functions remain largely unknown [6]. Prokaryotic AGOs are predicted to function as key components of a new class of defense systems against mobile genetic elements [6]. AGO proteins share two main structural features – the ∼140 residue central PAZ domain and the C-terminal 350-residue PIWI domain [1]. The PAZ domain contains an OB (oligonucleotide/oligosaccharide binding) fold, which is a typical single-stranded nucleic acid binding motif that has been shown to bind the 3′ end of short RNAs [7]. The PIWI domain shows extensive structural similarity to RNase H enzymes and is the catalytic center for rendering some AGOs that retain conserved residues able to target cleavage of RNA molecules complementary to AGO-bound small RNAs [8].

In most organisms investigated so far, which include *Drosophila*, the zebrafish and the mouse, the PIWI subfamily proteins play diverse function in germline-specific events and gametogenesis, where they bind PIWI-interacting RNAs (piRNAs) [5]. There is considerable evidence that the PIWI subfamily proteins (e.g. PIWI, AUB and AGO3) and piRNAs regulate transposon activity in *Drosophila* [9], [10], [11] but also in vertebrates [12], [13]. PIWI-type proteins have also a role in epigenetic function. The murine PIWI homologs, MILI and MIW2, are needed for the methylation of transposon-encoding genome regions [12], [13]. Furthermore, an epigenetic role has been attributed to PIWI along with piRNAs in *Drosophila* where it associates with chromatin and interacts directly with the heterochromatin protein 1 [14]. PIWI is also involved in the epigenetic control in somatic cells of the ciliate *Tetrahymena* where it is vital for the conjugative replication of macronucleus by removing highly repetitive transposon-like sequences [15].

*Leishmania* is a unicellular parasite, which is responsible for leishmaniasis. The parasite has two life stages, the invertebrate promastigote stage and the mammalian amastigote stage within the phagolysosomes of macrophages. *Leishmania* and related kinetoplastids regulate gene expression mainly posttranscriptionally [16]. Old World *Leishmania* species such as *L. infantum* and *L. major* lack a functional RNAi pathway [17], [18], [19]. The absence of typical Argonaute proteins in these parasites suggests their inability to suppress gene expression by RNAi [17]. Unlike the Old World *Leishmania* species, the New World species *L. braziliensis* (subgenus *Viannia*) code for RNAi pathway homologs [20] and can downregulate target genes by RNAi [21]. Similarly, the related trypanosomatid protozoan *Trypanosoma brucei* possesses a functional RNAi machinery and codes for an Argonaute 1 protein (AGO1), which can function as slicer [22]. This suggests that the RNAi machinery genes must have been lost independently in Old World *Leishmania* during evolution of the trypanosomatid lineage. Interestingly, while the RNAi-negative *L. major*/*L. infantum* strains [19]; http://tritrypdb.org) and *Trypanosoma cruzi* [23] have lost their AGO genes, they encode an Argonaute/PIWI*-*like protein homolog.

Here, we characterized a PIWI-like protein homolog in *Leishmania* and evaluated its putative function in regulating gene expression in these parasites. We show that genetic deletion of PIWI in both *L. infantum* and *L. major* species leads to an important growth defect of amastigotes *in vitro* and significantly delays disease pathology in mice. Furthermore, we demonstrate that unlike other canonical PIWI proteins in eukaryotes, the *Leishmania* PIWI-like protein resides mainly in the mitochondrion. As determined by a whole-genome DNA microarray analysis, loss of PIWI in *Leishmania* amastigotes affects mostly the expression of specific subsets of developmentally regulated genes, suggesting an important role of this protein in posttranscriptional regulation.

## Results

### The RNAi-negative Old World *Leishmania* Species Encode an Argonaute/PIWI-like Protein Lacking the Central PAZ Domain

**Figure 1:**
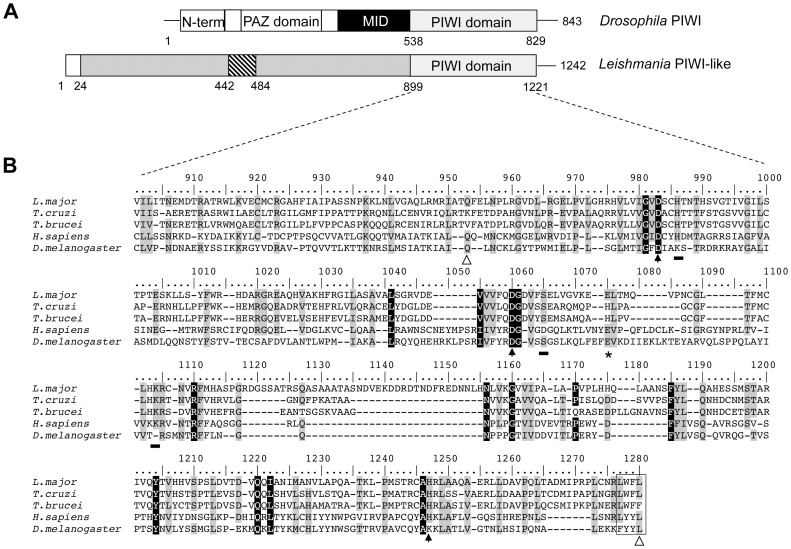
The *Leishmania* PIWI-like protein homolog forms a distinct group of the Argonaute/PIWI subfamily. (A) Structural domains (N-term, PAZ, MID, PIWI) of the Argonaute/PIWI proteins in higher eukaryotes (e.g. *Drosophila*) and in the unicellular parasitic protozoan *Leishmania*. In *Leishmania* only the C-terminal PIWI domain is found (residues 899–1221). The *Leishmania* PIWI-like homolog contains a small domain (residues 450–492, hatched box) with homology to the mitochondrial cytochrome C oxidase subunit VIIIb. A signal peptide of 24 residues has been predicted at the N-terminus of the *Leishmania* PIWI-like homolog. (B) Multiple sequence alignment of PIWI domains from *L. major*, the related trypanosomatids *Trypanosoma cruzi* and *T. brucei*, *H. sapiens* HIWI and the *Drosophila melanogaster* PIWI. The alignment was conducted using the Bioedit program [74]. Residues that are universally invariable among all eukaryotic Argonaute (AGO) proteins are shaded black. Residues that are highly conserved in all AGO proteins are shaded grey. Divalent anion-binding residues (Gln and Leu; residues 952 and 1242) are indicated by open arrows and the catalytic AGO triad (DDH; residues 981, 1048 and 1210) are indicated by black arrows. In the *Drosophila* PIWI homolog, the histidine (H) residue of DDH is not conserved but instead a lysine residue is present (DDK). The glutamate at position 1061, part of the RNase H-like DDE motif, is indicated by an asterisk. Residues putatively involved in RNA binding are underlined by a black rectangle. The C-terminus hydrophobic residues are boxed.

Argonaute (AGO) proteins found in eukaryotes are composed of four sections: N-terminal, PAZ domain, MID domain and PIWI domain [1], [24] (Figure 1A), while most archeal and prokaryotic AGOs do not contain the PAZ domain, and the domain architecture is variable [6]. The Argonaute/PIWI-like protein encoded by *Leishmania infantum* (LinJ.21.0470) and *L. major* (LmjF.21.0410) (http://tritrypdb.org) is a basic protein with a predicted MW of 133 kDa that is distinct from the canonical AGO/PIWI proteins in other eukaryotes. Indeed, the *Leishmania* PIWI-like homolog lacks the PAZ domain, which is typical of Argonaute proteins [4] but it has retained a PIWI-like C-terminal domain (from 899–1221 aa) (Figure 1A). Multiple sequence alignment of the *L. major* PIWI-like protein along with *Trypanosoma cruzi* (TcCLB.511367.240), *T. brucei* (Tb927.10.2220), and the *H. sapiens* (AAC97371) and *Drosophila* (AAF53043) PIWI homologs revealed a characteristic DDH motif composed of two aspartates and a histidine (Figure 1B), which constitutes a conserved catalytic core similar to the DDE (aspartate, aspartate, glutamate) catalytic triad of RNase H folding [25]. The *Leishmania* PIWI-like protein harbors also other invariable residues in all Argonaute proteins. These include conserved residues that define the PIWI and Argonaute subfamilies, the divalent cation-binding residues (Gln and Leu) for structural stability and catalytic activity and residues putatively involved in RNA binding [26], [27] (see Figure 1B).

The *L. major* and *L. infantum* PIWI-like proteins share 67% identity with *L. braziliensis* 1–1200 aa), 43% identity with *T. cruzi* (mainly from 581–1200 aa) and 41% identity with *T. brucei* (mainly from residues 580–1200). Interestingly, unlike the *T. cruzi Tc*PIWI homolog which comprises a full Argonaute architecture with an oligonucleotide/oligosaccharide binding (OB fold) motif at the N-terminus, a MID domain and a PIWI C-terminal domain [23], the *Leishmania* PIWI-like homolog has only retained the C-terminal PIWI domain. Unlike any other Argonaute protein, the *Leishmania* PIWI homolog contains a small domain at amino acid positions 450–492 with homology to the mitochondrial cytochrome C oxidase subunit VIIIb.

### The *Leishmania PIWI* Transcript is Preferentially Expressed in the Amastigote Life Stage of the Parasite

**Figure 2:**
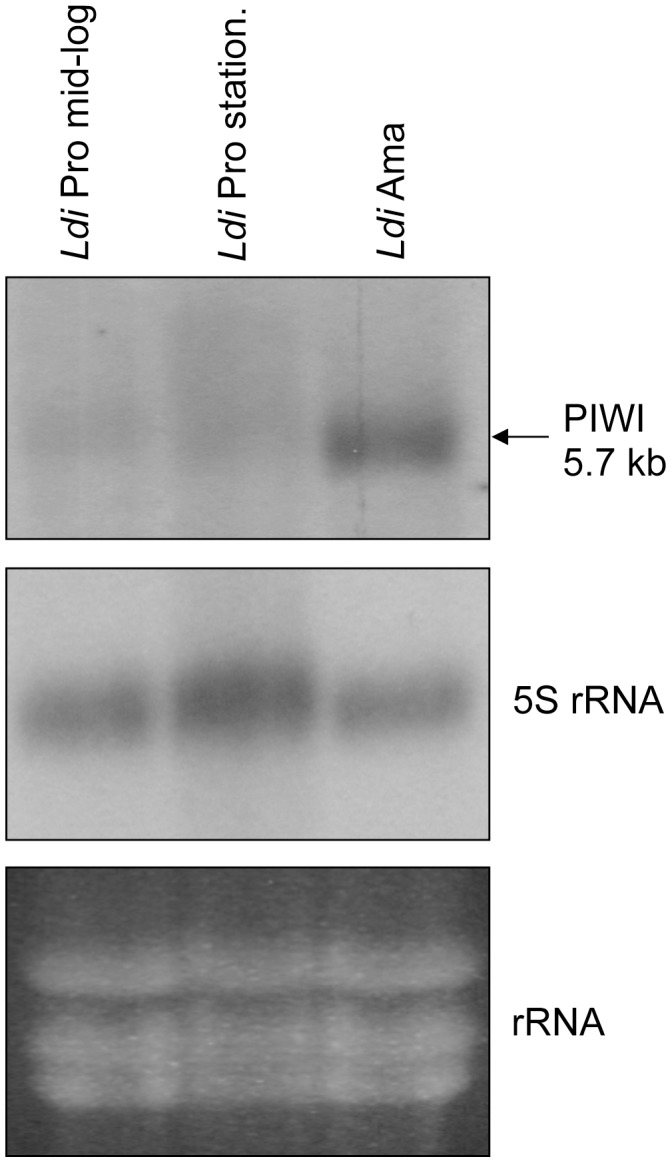
The *Leishmania PIWI* transcript is preferentially expressed in the amastigote lifestage. Northern blot hybridization to measure expression of the *L. infantum LinPIWI* transcript in exponentially- and stationary-grown promastigotes (Pro) and also in axenic amastigotes (Ama). The 5S rRNA probe was used for normalization of RNA loading. Northern blot experiments have been repeated two more times with identical results.

It has been reported in various organisms that PIWI protein has specific functions during different stages of growth. For example, MILI, a mouse homolog of PIWI, was shown to be involved in spermatogenesis [28]. Similarly, PIWI modulates cell division rates in *Drosophila* germline stem cells [29]. To determine *PIWI* transcript expression in both life stages of *L. infantum* we carried out northern blot analysis on total RNA from both promastigote and amastigote life stages using the *LinPIWI* ORF as a probe. Northern blot hybridization revealed that the level of *LinPIWI* transcript was preferentially expressed in axenic amastigotes compared to log-phase and stationary promastigotes (Figure 2). Interestingly, *L. major* DNA microarray experiments comparing mouse lesion-derived amastigotes to promastigotes revealed a differential *PIWI* expression in the amastigote stage (Natalia S. Akopyants, et al., in preparation; http://tritrypdb.org).

### The *Leishmania* PIWI-like Protein Resides Mainly in the Mitochondrion

**Figure 3:**
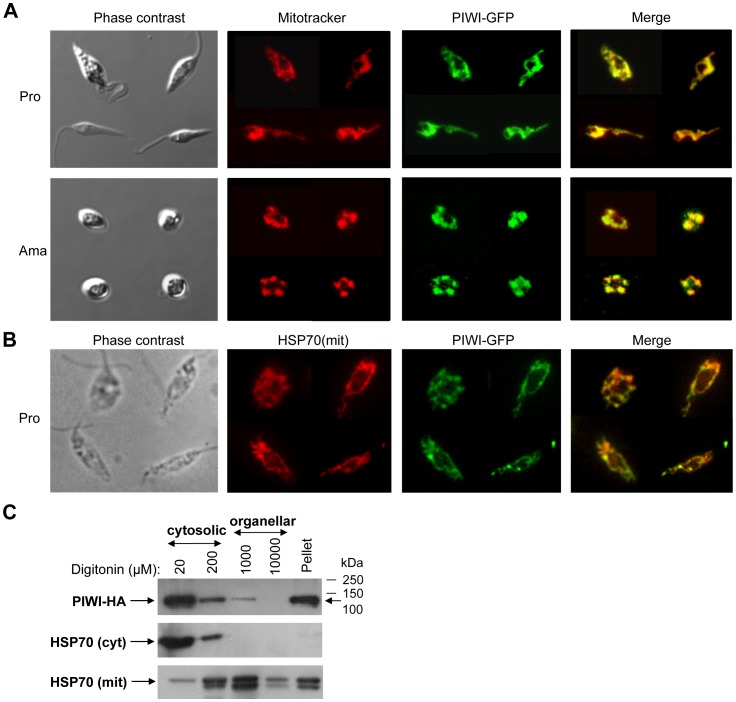
The *Leishmania* PIWI-like protein is mainly localized in the mitochondrion. (A) Localization of PIWI-GFP protein was monitored in both *L. infantum* promastigote (Pro) and axenic amastigote (Ama) forms by confocal microscopy. Mitotracker (a red fluorscent dye) was used to stain the *Leishmania* single mitochondrion. The green fluorescence signal of GFP was mostly co-localized with that of the Mitotracker. (B) Immunolocalization studies using epifluorescence microscopy to confirm mitochondrial localization of the PIWI-like protein. PIWI largely co-localizes with a known mitochondrial protein, HSP70. (C) Digitonin fractionation was carried out in *L. infantum Lin*HA-PIWI-HA recombinant promastigotes. Western blot of digitonin-fractionated samples (20 µM–10 mM) was probed with an anti-HA antibody to detect the PIWI protein and with anti-HSP70 (cytosolic) and anti-HSP70 (mitochondrial) antibodies used as controls for cytoplasmic and mitochondrial proteins, respectively. The 20 and 200 µM digitonin lanes correspond to cytosolic fractions and the 1 mM and 10 mM lanes correspond to organellar fractions. The pellet fraction contains membrane-associated proteins.

In higher eukaryotes, the PIWI subfamily proteins are localized in the cytosol or in the nucleus [5], [30]. Interestingly, the PIWI-like homolog in *Leishmania* possesses a putative mitochondrial targeting signal peptide at the N-terminus (1–24 aa) as suggested with the help of different servers that predict the presence and location of signal peptide cleavage sites in amino acid sequences from different organisms (e.g. SignalP 4.0, TargetP 1.1, PSORT and SOSUIsignal). Some of these softwares predict the sequence ARGECR (starting from amino acid 21) as the putative mitochondrial targeting signal peptide. MitoProt II-v1.101 analysis further asserted a possible targeting signal in the N-terminal 52 residues. To investigate whether the presence of a putative N-terminal mitochondrial targeting signal peptide could target the PIWI-like protein to the single mitochondrion of *Leishmania*, we fused the first 52 residues of the *L. infantum* PIWI-like protein to the GFP protein (designated as *Lin*N_52_PIWI-GFP; Figure S1A) and then stably transfected this vector into *L. infantum*. Epifluorescence microscopy showed that the N-terminal 52 residues were able to direct the GFP protein into the mitochondrion both in promastigotes and in amastigotes (Figure S1B). To assess the sub-cellular localization of the full-length *Leishmania* PIWI-like protein, the PIWI homolog was fused with GFP at the C-terminus (*Lmj*PIWI-GFP) and transfected into *L. infantum*. Confocal fluorescence microscopy showed that the PIWI-GFP protein was targeted specifically to the mitochondrion, both in promastigote and axenic amastigote forms (Figure 3A). To further confirm mitochondrial localization of the *Leishmania* PIWI-lke protein, we carried out co-localization studies with a known mitochondrial protein, HSP70. These studies clearly demonstrate that the PIWI-like protein largely co-localizes with the mitochondrial HSP70 protein (Figure 3B). Although most of the PIWI-lke protein in *Leishmania* seems to localize in the mitochondrion, there are some defined regions that did neither stain with Mitotracker nor co-localize with the mitochondrial HSP70 protein (Figure 3A–B), suggesting a partial cytosolic localization of this protein. Cellular compartmentalization by fractionation using various concentrations of digitonin (20 µM to 10 mM) confirms that part of the *Lin*PIWI-HA tagged protein (134 kDa) is enriched in the organellar fraction but it also demonstrates an important enrichment in the cytosolic fraction and the membrane-bound fraction (Figure 3C and data not shown). The enrichment of the *Lin*PIWI-HA protein in the pellet fraction, which represents total membrane fractions of the parasite, including the mitochondrial membrane, suggests that PIWI-like protein may be residing in the mitochondrial membrane. Interestingly, different softwares predicting the presence of membrane-spanning regions (e.g. TMpred, TopPred 0.01, PRED-TMR, HMMTOP, and SACS MEMSAT) revealed two short transmembrane helices in the *L. infantum* PIWI-like protein at positions 225–243 aa and 814–832 aa (data not shown), which may explain the membrane-association of this protein.

### The *Leishmania* PIWI-like Protein is not Involved in the Biogenesis of Small Non-coding RNAs

**Figure 4:**
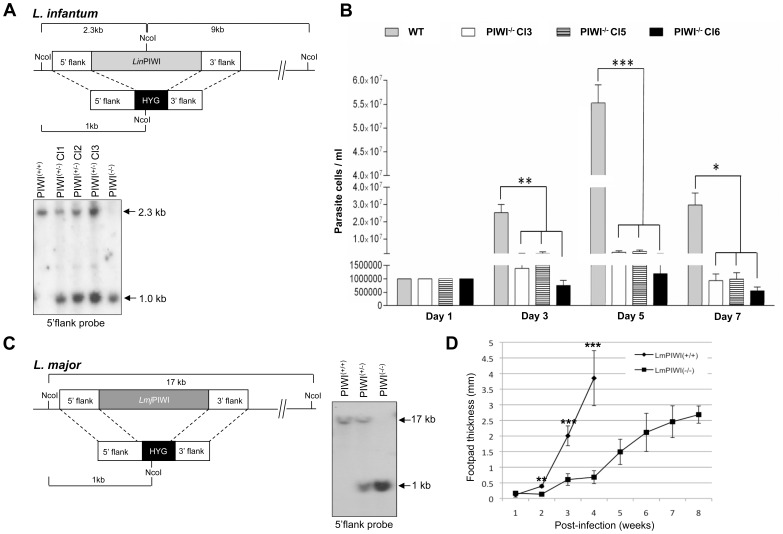
Genomic inactivation of the *PIWI* gene in *L. infantum* and *L. major* strains leads to a decrease in amastigote growth and disease pathology. (A, upper panel) Strategy to inactivate the *L. infantum PIWI* gene (*Lin*PIWI^(−/−)^) by genetic replacement. Both alleles of the *LinPIWI* single copy gene were replaced by the hygromycin phosphotransferase gene (*HYG*) by a loss of heterozygocity. (A, bottom panel) Southern blot hybridization of *L. infantum* genomic DNA digested with NcoI using the *PIWI* 5′ flank sequence as a probe. In *Lin*PIWI^(+/+)^, only a 2.3 kb band which corresponds to the wild type alleles was detected. In *Lin*PIWI^(+/−)^ clones (C1, C2 and C3), in addition to the wild type allele, one more band of 1.0 kb (for the *HYG* gene integration) was detected. In *Lin*PIWI^(−/−)^, only one band of 1.0 kb (for the *HYG* gene replacement) was detected but not the 2.3 kb band. (B) Growth curve of *L. infantum* axenic amastigotes for *Lin*PIWI^(+/+)^ and *Lin*PIWI^(−/−)^ independent clones 3, 5 and 6. The growth pattern of *L. infantum* WT and *Lin*PIWI^(−/−)^ clones on days 1, 3, 5 and 7 was analyzed by one-way ANOVA followed by a Tukey’s post-test using GraphPad Prism (version 3.03) software. Significant differences between the various groups are indicated (*, *P*<0.05; **, *P<*0.01; and ***, *P*<0.001). (C, left panel) Strategy to generate a PIWI null mutant in *L. major* (*Lmj*PIWI^(−/)^). The *LmjPIWI* alleles were replaced by the *HYG* expression cassette by a single round of gene targeting. (C, right panel) Southern blot hybridization using the *L. major PIWI* 5′ flank sequences as a probe. The 17 kb band corresponds to the *PIWI* wild type alleles and the 1.0 kb band corresponds to the *HYG* gene integration into the *PIWI* genomic locus. (D) *L. major* infection rates in mice estimated by measuring the size of footpad lesions at different time points post-infection in BALB/c mice infected with either the wild type (*Lmj*PIWI^(+/+)^) strain or the *Lmj*PIWI^(−/−)^ null mutant (clone 1). Control mice were sacrificed at 4 weeks post-infection due to the larger size of the lesions. Data shown here are the mean±SD of 6 mice per group and are representative of three independent experiments. The footpad thickness of *L. infantum* WT and *Lmj*PIWI^(−/−)^ null mutant on weeks 1, 2, 3 and 4 was analyzed by t-test (non parametric) using GraphPad Prism (version 3.03) software. Significant differences between WT and *Lmj*PIWI^(−/−)^ are indicated (**, *P<*0.01 and ***, *P*<0.001).

The evolutionarily conserved Argonaute/PIWI family of proteins are crucial for the biogenesis and function of the 24–32 nucleotide long piRNAs that are generated from long single-stranded RNA precursors often encoded by repetitive intergenic sequences in the genome [5]. As PIWI proteins do have the RNase H fold and some PIWI subfamily proteins have shown slicer activity, it is possible that they cleave the precursor into mature piRNAs [5]. The *Leishmania* genome is full of repetitive sequences within intergenic regions and 3′UTRs such as the highly structured SIDER retroposon elements [31], [32], which may be a substrate for PIWI. To assess the putative role of the *Leishmania* PIWI-like protein in the production of small non-coding RNA species, we engineered a *L. infantum* mutant lacking the *PIWI* gene (*Lin*PIWI^(−/−*)*^) by gene replacement through homologous recombination. Both alleles of the *PIWI* single copy gene were replaced by the hygromycin (*HYG*) resistance marker gene by loss of heterozycocity as revealed by Southern blot analysis using the 5′-flank region of *PIWI* as a probe (Figure 4A). The integration of the *HYG* cassette into both *Lin*PIWI alleles was confirmed by the presence of the ∼1 kb NcoI hybridizing band (Figure 4A upper panel and data not shown) and the absence of the 2.3 kb NcoI fragment corresponding to the wild type *LinPIWI* alleles (Figure 4A, bottom panel).

To evaluate if there is any change in the small RNA species population between the *Lin*PIWI^(−/−)^ mutant and wild type, we enriched for small RNAs (≤200 nt) (see Materials and Methods) that were labelled with [γ-^32^P]ATP and resolved on 15% urea acrylamide gel. This experiment revealed no differences between the *Lin*PIWI^(−/−)^ mutant and wild type in the pattern of small or microRNAs derived from either SIDER elements [31] and or other type of noncoding RNA species previously described in our laboratory [33] (Figure S2A and data not shown). It was previously reported that the mouse MIWI protein and piRNAs co-fractionate with polysomes, indicating a potential role of these components in translational control [34]. Therefore, small RNA species of the wild type and *Lin*PIWI^(−/−)^ mutant were enriched from RNPs, 40S and 60S subunits, 80S monosome and polysomal fractions following 15–45% sucrose gradient fractionation. As also seen with the total small RNA population, there were no notable differences in small RNA species associated with ribosomes between the wild type and *Lin*PIWI^(−/−)^ mutant (Figure S2B), hence suggesting that *Lin*PIWI is most likely not involved in the biogenesis or the stability of small RNAs in *Leishmania*.

Mitochondrial localization of the *Leishmania* PIWI-like protein prompted us also to evaluate if PIWI through its association with small guide RNAs plays a role in site-specific edition of mitochondrial mRNAs, a posttranscriptional addition or deletion of uridine residues that takes place exclusively within the mitochondrion of trypanosomatid protozoa [35]. We therefore examined the cytochrome C oxidase subunit II (COXII) and cytochrome B (CYB) transcripts, known to be extensively edited in kinetoplastids [36]. The editing pattern of *COXII* (Figure S3A) and *CYB* (Figure S3B) transcripts in *Lin*PIWI^(−/−)^ was identical to that of wild type, suggesting that *Lin*PIWI is not involved in RNA editing. Furthermore, we evaluated if *Lin*PIWI plays any role in kinetoplastid DNA (kDNA) replication within the mitochondrion [37]. However, isolation of the kDNA from wild type and *Lin*PIWI^(−/−)^ strains showed no differences in kDNA pattern (Figure S4).

### Genomic Depletion of the PIWI-like Protein Significantly Delays *Leishmania* Amastigote Growth and Disease Pathology in Mice

Next, we assessed the effect of PIWI depletion on parasite growth both in vitro and in vivo. The *L. infantum* PIWI^(−/−)^ mutant did not show any growth defect when cultured as promastigotes (data not shown). However, growth of several independent clones lacking PIWI as axenic amastigotes was significantly delayed compared to the wild type (Figure 4B). To evaluate the role of PIWI in amastigote intracellular survival and disease development, we inactivated the *PIWI* gene in *L. major* (*Lmj*PIWI^(−/−)^) (Figure 4C) as described above for *L. infantum* (Figure 4A). PCR analysis (data not shown) and Southern blot hybridization (Figure 4C) confirmed the inactivation of *LmjPIWI* gene, as determined by the absence of the ∼17 NcoI kb band corresponding to the wild type alleles and the detection of the ∼1 kb NcoI *HYG* integration band. An equal number of stationary-phase wild type (*Lmj*PIWI^(+/+)^) and *Lmj*PIWI^(−/−)^ mutant (clone 1) were injected into the footpad of BALB/c mice and infection was monitored for up to 8 weeks by measuring the size of the footpad lesions. Three independent experiments confirmed a significant delay in lesion development in mice infected with *Lmj*PIWI^(−/−)^ in comparison to the wild type (*Lmj*PIWI^(+/+)^)-infected animals, even at 8 weeks post-infection (Figure 4D). Moreover, an independent experiment using a different *Lmj*PIWI^(−/−)^ clone (clone 3) showed a similar delay in lesion development than clone 1 (data not shown). All our attempts to complement the PIWI^(−/−)^ null mutant phenotype failed as none of the episomally expressed PIWI epitope-tagged (e.g. HA, Myc, and GFP) proteins or even the original *PIWI* gene without any tag (Figure S5) transfected into *Lmj*PIWI^(−/−)^ or *Lin*PIWI^(−/−)^ mutant background could rescue efficiently for the loss of the endogenous PIWI protein (data not shown). The fact that we could only detect the PIWI-tagged proteins by western blot after enrichment or cell fractionation (Figure 3C and data not shown) suggests that the levels of the ectopically expressed PIWI protein might be too low or not properly regulated outside its genomic context. We cannot also exclude that the episomal vectors used for rescuing the mutant phenotype were lost in the absence of drug selection in mice. Nevertheless, it is important to highlight that we could observe a similar defect in the PIWI^−/−^ mutant amastigote growth in two distinct *Leishmania* species and for different clones, which further supports the specificity of the role of PIWI protein in the amastigote growth of the parasite.

### Loss of the *L. infantum* PIWI-like Protein Affects the Expression of Specific Subsets of Stage-regulated Transcripts

**Figure 5:**
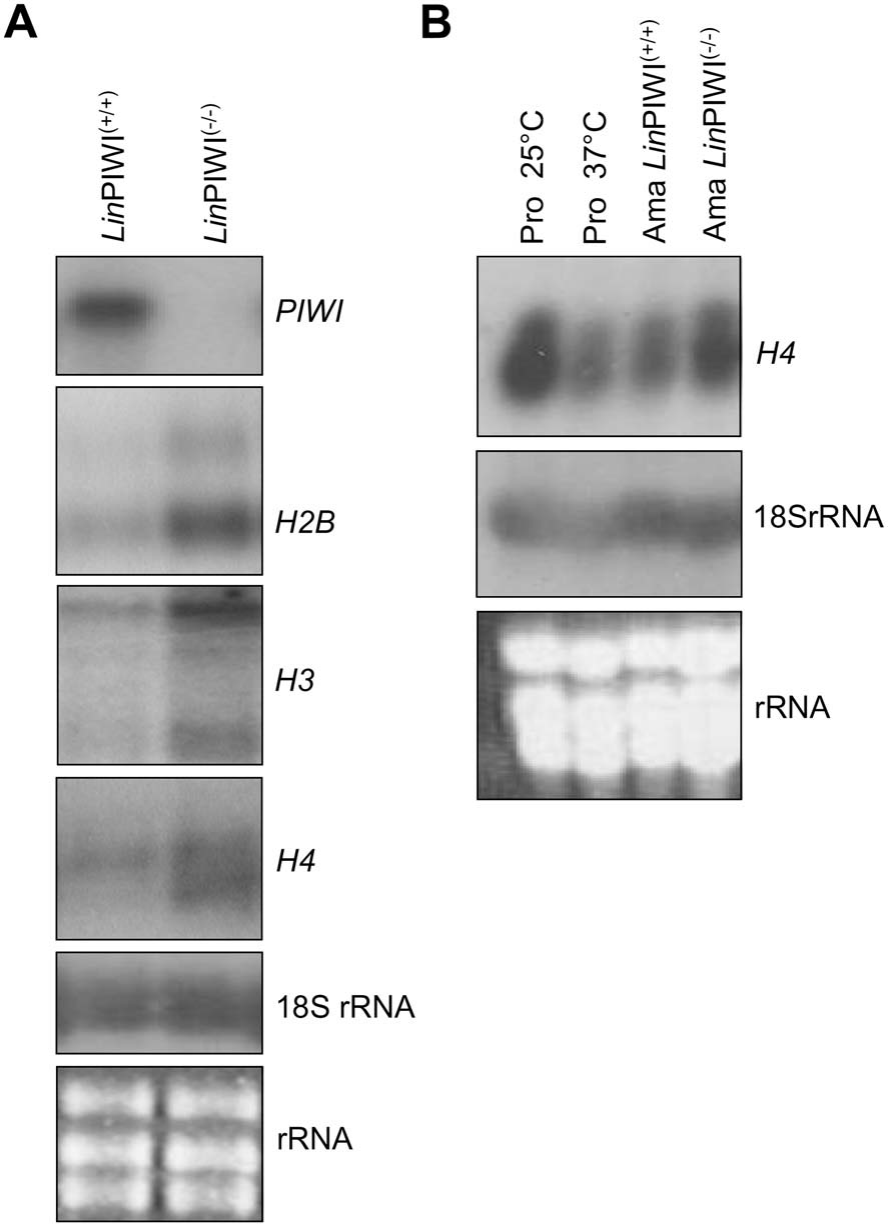
The histone mRNAs are upregulated in the *L. infantum* PIWI−/− null mutant. (A) Northern blot hybridization of total RNA isolated from wild type *Lin*PIWI^(+/+)^ and *Lin*PIWI^(−/−)^ axenic amastigotes to evaluate changes in the accumulation of the histone *H2B* (LinJ.19.0030), *H3* (LinJ.10.0920) and *H4* (LinJ.21.0020) transcripts. Hybridization was carried out with radiolabeled *PIWI* ORF and histone ORF probes. There are two bands hybridizing with the *H2B* and *H3* gene probes as reported previously [41], [43]. (B) Northern blot hybridization to confirm upregulation of the *H4* transcript in *Lin*PIWI^(−/−)^ amastigotes or in heat-stressed promastigotes compared to unstressed parasites. The 18S RNA blot hybridization and EtBr-stained gels were used as loading controls.

To determine whether PIWI depletion could affect expression levels of specific mRNA subsets, we performed a whole-genome DNA microarray analysis to compare gene expression profiles between *L. infantum* wild type (*Lin*PIWI^(+/+)^) and *Lin*PIWI^(−/−)^ null mutant amastigotes. For this experiment, we used a previously described high-density 70-mer oligonucleotide *Leishmania* genome microarray [38]. After subtracting the background, the difference of 1.7-fold in the signal intensity between the experimental conditions used for a given gene was chosen as the cut-off, provided that the *p* value confidence was higher than 95%. The most significant differences in gene expression between *L. infantum* wild type and *Lin*PIWI^(−/−)^ null mutant are presented in Table 1. Several membrane-bound protein encoding transcripts seem to be downregulated in the *Lin*PIWI^(−/−)^ mutant, including the major surface protein GP63 leishmanolysin, the surface antigen-like protein, the membrane-bound acid phosphatase 2 and several transporters. The list of the downregulated genes includes also transcripts coding for protein kinases and hypothetical conserved proteins. Transcripts upregulated in the *Lin*PIWI^(−/−)^ mutant code mainly for histones. Histone *H1*, *H2A*, *H2B*, *H3* and *H4* transcripts were upregulated by 2-fold in average in the *Lin*PIWI^(−/−)^ mutant (Table 1). The accumulation of histone transcripts in *Lin*PIWI^(−/−)^ was further confirmed by northern blot analysis (Figure 5A). It was previously described [39], [40], [41], [42], [43] and shown also here that histone mRNAs in *Leishmania* are generally downregulated in the amastigote stage or when subjected to heat stress (Figure 5B).

**Table 1:** Genes differentially expressed between *Leishmania infantum* wild type and *Lin*PIWI^(−/−)^ axenic amastigotes as assessed by DNA microarray analysis.

Accession number (TriTryp DB)/Biological process^a^	Gene description	Fold difference (Ax. Ama/Pro)	*p* value	Stage-specific gene expression^b^
**Downregulated genes**				
**Surface membrane proteins**				
LinJ.04.0200	surface antigen-like protein	0.5824	4,32E−04	Amastigote
LinJ.05.0900	surface antigen-like protein	0.5561	4,58E−04	Amastigote
LinJ.36.2720	membrane bound acid phosphatase 2, putative	0.5375	2,59E−05	Promastigote
LinJ.33.0310	glucose transporter/membrane transporter D2, putative	0.4638	4,20E−05	Amastigote
LinJ.07.1340	amino acid transporter, putative (AAT19)	0.4731	1,22E−05	Amastigote^c^
LinJ.10.0490, LinJ.10.0500, LinJ.10.0510, LinJ.10.0520, LinJ.10.0530	GP63, leishmanolysin	0.5641	4,20E−04	Promastigote
LinJ.28.0600, LinJ.28.0610	major surface protease GP63, putative, leishmanolysin, putative	0.5409	2,59E−05	Promastigote
LinJ.10.1450	pteridine transporter, putative	0.5859	2,87E−04	Amastigote
LinJ.19.0870	folate/biopterin transporter, putative	0.4271	5,65E−05	Promastigote
**Lipid metabolic process**				
LinJ.14.0710^d^, LinJ.14.0720^d^, LinJ.14.0730, LinJ.14.0740, LinJ.14.0760*	fatty acid elongase, putative	0.3810	9,45E−06	Amastigote
LinJ.23.1560	lathosterol oxidase-like protein	0.5687	1,50E−05	Promastigote
**Cell motility**				
LinJ.16.0920, LinJ.16.0930	flagellar calcium binding protein, putative	0.5921	7,57E−04	Amastigote
**Cell communication**				
LinJ.17.0130	receptor-type adenylate cyclase, putative	0.5439	4,32E−04	Amastigote
**Protein modification**				
LinJ.08.0670*	protein kinase, putative	0.5228	2,95E−05	Amastigote
LinJ.04.1230	protein kinase, putative, casein kinase I, putative	0.5050	2,93E−04	Amastigote
LinJ.36.2420	protein kinase, putative, serine/threonine protein kinase, putative	0.5683	2,48E−03	Amastigote
LinJ.27.1680	casein kinase I-like protein	0.5586	8,69E−05	Amastigote
**Cell cycle**				
LinJ.30.3690	CYC2-like protein, putative	0.5706	5,48E−04	Amastigote
**Unclassified**				
LinJ.18.0080	hypothetical protein, conserved	0.4548	2,59E−05	Amastigote
LinJ.18.0140*	hypothetical protein, conserved	0.5865	9,24E−04	Constitutive
LinJ.19.0560*	hypothetical protein, conserved	0.4814	5,65E−05	Promastigote
LinJ.19.0570*	hypothetical protein, conserved	0.4814	5,65E−05	Amastigote
LinJ.06.1360*	hypothetical protein, conserved	0.4125	2,32E−04	Amastigote
LinJ.10.0280*	hypothetical protein, conserved	0.5471	4,25E−04	Promastigote
LinJ.07.0850	hypothetical protein, unknown function	0.4690	1,69E−04	Amastigote
LinJ.36.0630	hypothetical protein, unknown function	0.5452	6,11E−04	Amastigote^c^
LinJ.23.0700*	hypothetical protein	0.5694	1,78E−04	Constitutive
LinJ.23.1190	hypothetical protein, unknown function	0.5340	3,33E−04	Promastigote
LinJ.26.2710	hypothetical protein, unknown function	0.5522	8,69E−05	Amastigote
LinJ.27.0960*	hypothetical protein, conserved	0.5425	4,93E−04	Promastigote
LinJ.29.2940	hypothetical protein, conserved/RNA bndingprotein RBP6	0.3800	1,22E−05	Promastigote
LinJ.31.0750*	hypothetical protein, conserved/Major facilitator superfamily	0.4655	9,55E−05	Amastigote
LinJ.36.4050*	similar to *L. major* 1411.4-like protein	0.5921	2,93E−04	Amastigote
LinJ.21.0470	argonaute-like protein, putative, PIWI-like protein 1, putative	0.3278	3,21E−03	Amastigote
**Upregulated genes**				
**Nucleosome assembly**				
LinJ.27.1070	histone H1, putative	1.8331	2,97E−03	Amastigote
LinJ.27.1120	histone H1, putative	1.8331	2,97E−03	Amastigote
LinJ.21.1160, LinJ.21.1170	histone H2A	1.6954	9,88E−04	Promastigote
LinJ.29.1850, LinJ.29.1860, LinJ.29.1870	histone H2A, putative	1.6954	9,88E−03	Promastigote
LinJ.09.1410	histone H2B	2.2143	4,83E−04	Promastigote
LinJ.17.1320	histone H2B	2.2143	4,83E−04	Promastigote
LinJ.19.0030, LinJ.19.0040	histone H2B	2.2143	4,83E−04	Promastigote
LinJ.10.0920	histone H3	1.9454	6,30E−04	Promastigote
LinJ.10.1050, LinJ.10.1070	histone H3	1.9454	6,30E−04	Promastigote
LinJ.16.0600, LinJ.16.0610	histone H3, putative	1.9454	6,30E−04	Promastigote
LinJ.31.3320	histone H4	2.2006	1,51E−04	Promastigote
LinJ.15.0010	histone H4	2.2006	1,51E−04	Promastigote
LinJ.25.2560	histone H4	2.2006	1,51E−04	Promastigote
LinJ.06.0010	histone H4	2.2006	1,51E−04	Promastigote
LinJ.35.0020	histone H4, putative, pseudogene	2.2006	1,51E−04	Promastigote
LinJ.35.1320	histone H4	2.2006	1,51E−04	Promastigote
LinJ.36.0020	histone H4	2.2006	1,51E−04	Promastigote
LinJ.21.0020	histone H4	2.2006	1,51E−04	Promastigote
**GPI anchor biosynthetic process**				
LinJ.12.0140*	Alg9-like mannosyltransferase, putative	1.6925	1,51E−03	Constitutive
**Translation**				
LinJ.26.0150, LinJ.26.0160	60S ribosomal protein L7, putative	1.7545	1,85E−04	Constitutive
**Energy metabolism**				
LinJ.26.0450*	ATPase subunit 9, putative	1.8391	6,26E−03	Constitutive
**Unclassified**				
LinJ.35.1470	hypothetical protein, conserved	1.9454	1,75E−04	Constitutive
LinJ.06.0920	hypothetical protein, conserved	2.1498	1,43E−02	Constitutive
LinJ.08.0440	hypothetical protein, conserved	2.5012	5,59E−03	Amastigote
LinJ.16.0570	hypothetical protein, conserved	2.5899	2,67E−03	Constitutive

a Gene functions are based on Gene Ontology (GO) annotation. The main categories are shown here.

b Stage-specific gene expression of the modulated transcripts in the *L. infantum Lin*PIWI^(−/−)^ mutant is based on the data by [38], [44].

c There is some discrepancy between the intracellular and axenic amastigote microarray data. These genes seem to be more expressed in promastigotes when comparing *L. infantum* intracellular amastigotes to promastigotes.

d A probe recognizing LinJ.14.0710, LinJ.14.0720¸ LinJ.14.0730, LinJ.14.0740 and LinJ.14.0760 showed that theses genes were more expressed in the amastigote stage. Another probe recognizing LinJ.14.0710 and LinJ.14.0720 showed that theses genes were more expressed in the promastigote stage.

* Genes encoding proteins with one to several transmembrane domains.

Another intriging finding from the DNA microarray analysis is that the vast majority of the modulated transcripts in the *Lin*PIWI^(−/−)^ mutant has been reported previously to be differentially expressed in either lifestage of the parasite [44] (Table 1). Amongst the downregulated genes, the majority seems to be more expressed in amastigotes (24 amastigote-specific vs. 10 promastigote-specific) (Table 1). In the case of the upregulated genes, the large majority is more expressed in promastigotes (16 promastigote-specific vs. 3 amastigote-specific) (Table 1).

### *Leishmania* Amastigotes Lacking PIWI Exhibit a Higher Sensitivity to Apoptosis-like Cell Death Inducing Agents

**Figure 6:**
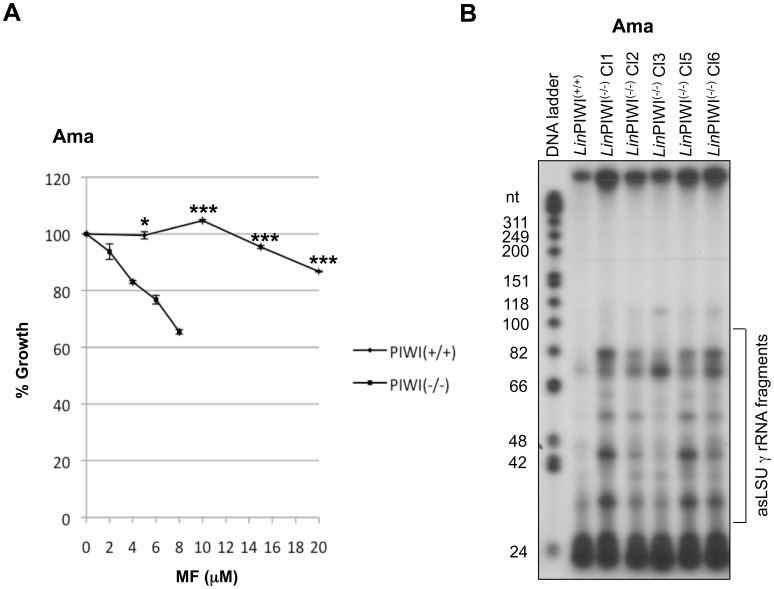
*Leishmania* amastigotes lacking PIWI are more sensitive to apoptosis-like cell death inducing agents. (A) *Lin*PIWI^(−/−)^ was shown to be highly sensitive to miltefosine (MF). Equal number of *L. infantum* axenic amastigotes (Ama) of wild type and *Lin*PIWI^(−/−)^ mutant strains were treated with various concentrations of MF for a period of 8 hrs. Drug sensitivity was evaluated by measuring the OD at 600 nm in a 96 well plates. The results are expressed as mean ± SD of biological triplicates and the experiments were conducted twice with similar results. The comparison of growth percentage between *L. infantum* WT treated with 0, 5, 10, 15, 20 µM of MF and *Lin*PIWI^(−/−)^ treated with 0, 2, 4, 6 and 8 µM of MF, respectively (*Lin*PIWI^(−/−)^ is much more sensitive to MF than WT) was analyzed by t-test (non parametric) using GraphPad Prism (version 3.03) software. Significant differences between WT and *Lin*PIWI^(−/−)^ are indicated (*, *P<*0.05; ***, *P*<0.001). (B) Primer extension analysis of *L. infantum* wild type (*Lin*PIWI^(+/+)^) and five independent clones (Cl 1–3, Cl 5 and Cl 6) of *Lin*PIWI^(−/−)^ null mutant grown as axenic amastigotes using a forward primer corresponding to nucleotides 101–118 of the sLSU γ rRNA to detect antisense (as) LSU γ rRNA fragmentation.

*Leishmania* undergoes various steps of apoptosis-like cell death following treatment with anti-leishmanial drugs such as trivalent antimony, miltefosine, and or exposure to H_2_O_2_ [45], [46], [47]. Since the *Leishmania* PIWI-like protein is mainly localized in the mitochondrion and mitochondria are known to be central for apoptosis-associated deregulation [48], we investigated whether sensitivity to apoptosis was altered in the *Lin*PIWI^(−/−)^ mutant. Indeed, exposure of the wild type and *Lin*PIWI^(−/−)^ mutant to increasing concentrations of miltefosine (MF) showed that parasites lacking PIWI were more sensitive to MF than the wild type cells (Figure 6A). We have reported recently that induction of apoptosis by MF triggers fragmentation of antisense (as) rRNA, which leads to rRNA degradation [49]. Since the *Lin*PIWI^(−/−)^ mutant is more sensitive to MF, we tested whether asrRNA fragmentation was increased in the PIWI null mutant as compared to the wild type cells. Primer extension analysis to specifically detect antisense RNA complementary to the large subunit gamma (LSU γ) rRNA, one of the six 28S rRNA processed fragments in *Leishmania,* revealed significantly higher fragmentation of the asLSU γ rRNA in the *Lin*PIWI^(−/−)^ mutant compared to the wild type (Figure 6B). Together, these results suggest a protective role of the *Leishmania* PIWI-like protein against apoptosis.

## Discussion

Although Argonaute/PIWI proteins are conserved between species, they have undergone remarkable structural evolution and functional diversification. In this study, we show that unlike higher eukaryotes, *Leishmania* encodes a distinct homolog of AGO/PIWI-like proteins which possesses a PIWI domain harboring the conserved DDH catalytic triad but lacks the typical PAZ domain involved in the sequence-independent binding of ssRNA [4]. Thus, similarly to some prokaryotic AGO proteins [6], the N-terminal part of the *Leishmania* AGO/PIWI protein including PAZ domain was lost independently in several lineages. Interestingly, the recently characterized PIWI-like homolog from the related trypanosomatid *T. cruzi* exhibits a higher domain architecture similarity to the AGO/PIWI proteins of higher eukaryotes [23] than to the *Leishmania* PIWI-like protein. However, even, in *T. cruzi,* the PAZ domain is divergent from the canonical AGO proteins, suggesting that AGO/PIWI-like proteins in trypanosomatids devoided RNAi [22] may have other unknown functions unrelated to RNAi.

Functional analysis of the *Leishmania* PIWI-like protein revealed its important role in the disease-causing amastigote stage of the parasite. Genetically engineered *L. infantum* and *L. major* mutants lacking the *PIWI* gene demonstrated a significant growth defect as amastigotes both *in vitro* and in an experimental mouse system. These data are consistent with a higher expression of the *PIWI* transcript in amastigotes. The stage-specific expression of PIWI is well documented in higher organisms. In mammals, PIWI protein is specifically expressed in the testes of male during the germ cell development. In male mice, absence of PIWI homologs (MIWI, MILI and MIWI2) leads to the arrested spermatogenesis, subsequent apoptosis of the germ cells and eventual male sterility whereas in female, MIWI, MILI and MIWI2 knockout mice are fertile and produce fertile off springs [50], [51]. These piRNAs along with PIWI proteins are well known for their role in regulating transposon activity both in invertebrates (e.g. *Drosophila* and *C. elegans*) [52], [53] and in vertebrates (zebrafish) [54], thereby maintaining genomic integrity. The *Leishmania* genome is largely invaded by short interspersed degenerate retroposon elements (SIDERs), which are mainly located within 3′UTRs and intergenic regions and seem to play important roles in posttranscriptional regulation of gene expression [31], [32]. Therefore, we hypothesized that PIWI might be involved in the control of these widespread repetitive elements in the *Leishmania* genome. However, all our attempts to isolate small RNAs associated with PIWI derived either from the repetitive SIDER elements or from other type of noncoding RNA species identified previously in *Leishmania* [31], [33] failed, which excludes any major role of this protein in the biogenesis of small RNAs. This is in agreement with the absence of RNAi in Old World *Leishmania* species [17].

Here, we show that the *Leishmania* PIWI-like protein plays a role in the regulation of specific subsets of developmentally regulated transcripts in the two lifestages of the parasite. Indeed, most of the downregulated genes in the *Lin*PIWI^(−/−)^ mutant are preferentially expressed in amastigotes whereas the vast majority of the upregulated genes are differentially expressed in promastigotes. This suggests that *Lin*PIWI is involved either directly or indirectly in the stabilization of subsets of amastigote-specific transcripts and destabilization of promastigote-specific transcript subsets. In *Drosophila*, it has been shown that the piRNA pathway is involved in the decay of maternal messenger RNAs and in translational repression [55]. How does *Lin*PIWI protein modulate the expression of these specific subsets of mRNAs is not yet understood. It is really intriguing that many of the downregulated genes in the *Lin*PIWI^(−/−)^ mutant code for membrane-bound proteins and that the majority of the upregulated genes encode histones. Amongst the downregulated transcripts encoding surface or membrane-bound proteins some have been reported to contribute to parasite virulence. For example, the major surface protease GP63 plays a key role in the interaction of the parasite with its mammalian host and its survival within macrophages [56]. Glucose transporter deficient *Leishmania* exhibit higher sensitivity to oxidative stress and are not viable in the disease-causing amastigote stage of the parasite [57], [58]. Amino acid transporters have also been found to be important for intracellular amastigote growth [59] or to improve virulence [60]. Differences in the expression of surface proteins or essential transporters may impact on the parasite’s intracellular survival as corroborated by the decreased infectivity of the PIWI^(−/−)^ mutant in mice. In contrast to transcripts encoding surface or membrane-bound proteins, the histone mRNAs were unregulated by ∼2-fold in the amastigote stage of the *L. infantum* PIWI ^(−/−)^ mutant. In *Leishmania*, *H2A, H2B, H3* and *H4* transcripts are generally expressed at higher levels in promastigotes than in amastigotes [39], [40], [41], [42], [43]. As histones are an integral part of nucleosomes, the basic unit of chromatin, it is possible that PIWI plays a role in epigenetic control in *Leishmania,* similarly to what has been reported in higher eukaryotes [5]. Indeed, a positive epigenetic role of PIWI along with its associated piRNAs has been attributed to the heterochromatic activation through histone modifications, although PIWI has a global function in silencing of heterochromatin [61]. To the best of our knowledge this is the first time that an inverse relationship between an Argonaute/PIWI protein and regulation of histone mRNAs is reported. It is interesting to mention that *Leishmania* amastigotes express higher levels of the *PIWI* transcript and lower levels of histone transcripts whereas promastigotes express low levels of *PIWI* transcript but higher levels of histones, suggesting that PIWI directly or indirectly negatively regulates stability of histone mRNAs.

Unlike other eukaryotes where PIWI proteins localize in the nucleus as epigenetic regulators and regulators of transposon activity [5], [11], [13], [28], [54], [62] or in the cytoplasm as regulators of RNA stability and translation [30], [34], [63], [64], the *Leishmania* PIWI-like protein is mainly localized in the single mitochondrion of the parasite. Targeting of the *Lin*PIWI-like protein into the mitochondrion occurs most likely via its N-terminal signal targeting peptide. In contrast to *Leishmania*, the *T. cruzi* PIWI protein (*Tc*PIWI) was shown to reside solely in the cytoplasm [23]. This difference may be attributed to the poor conservation of the N-terminal amino acid sequence of *Tc*PIWI compared to *Lin*PIWI. The enrichment of *Lin*PIWI protein in the membrane-bound protein fraction and the presence of two predicted short transmembrane helices within the PIWI protein suggest localization in the mitochondrial outer membrane. This brings up the interesting possibility that *Lin*PIWI might be exposed to the cytoplasmic side of the outer mitochondrial membrane, thus allowing it to interact with cytosolic proteins and to contribute to the stability or translation of specific subsets of mRNAs as shown by DNA microarray experiments. Despite its mitochondrial localization, the *Leishmania* PIWI-like protein does not seem to be involved in RNA editing [35] or in kinetoplast DNA (kDNA) replication [37], two processes that take place exclusively in the mitochondrion of these parasitic protozoa. Thus, the mitochondrial function of PIWI in *Leishmania* remains elusive.

Given that the *Leishmania* PIWI-like protein preferentially localizes into the mitochondrion and that mitochondria play a key role in cell viability, we also addressed the putative role of PIWI in apoptosis-like programmed cell death, which can be induced by various leishmanicidal agents. In higher organisms, PIWI is known to be involved in apoptosis. For example, in zebrafish and mice the loss of PIWI homologs triggers apoptosis in the germ cells [28], [54]. High levels of the PIWI family protein PIWI2 have been detected in various tumors where it acts like an oncogene that activates various signal transduction cascades that prevent apoptosis [65]. Thus, PIWI protein is guarding tumor cells from apoptosis. Interestingly, the *Lin*PIWI^(−/−*)*^ null mutant exhibits high sensitivity to the apoptosis inducing agent miltefosine. It is possible that in the absence of PIWI the mitochondrial membrane may be destabilized, rendering hence the parasite more prone to apoptosis inducing agents. Overall, our work demonstrates that the *Leishmania* PIWI-like protein homolog exhibits novel features that are distinct from other well-characterized members of the AGO/PIWI protein family in higher eukaryotes. Future studies will shed more light into the putative function of this divergent PIWI homolog on RNA regulation and amastigote development.

## Materials and Methods

### *Leishmania* Culture and Mouse Infection

*L. infantum* MHOM/MA/67/ITMAP-263 and *L. major* LV39 MRHO/SU/59/P strains and have been described previously [38]. Promastigotes were cultured at 25°C in SDM-79 medium pH 7.0 supplemented with 10% heat-inactivated FCS (Wisent) and 5 mg/ml of hemin. To differentiate *L. infantum* promastigotes into axenic amastigotes, late-stationary promastigotes were inoculated in MAA-20 medium supplemented with 20% FCS and grown at pH 5.6 and 37°C in a 5% CO_2_ atmosphere, as reported previously [66]. Fully differentiated amastigotes remained stable in culture and were used after 2–3 passages. For the *in vivo* studies, 1×10^7^ *L. major* wild type and *L. major* PIWI^(−/−)^ stationary promastigotes were used to infect female BALB/c mice subcutaneously in the footpad and cutaneous lesions were followed for up to 6–8 weeks post-infection. The infection protocol in mice has been approved by the animal protection committees of the CHUQ Research Centre (CPA-CHUQ) and Laval University (CPAUL) (authorization number: 2009148-3). *L. infantum* axenic amastigotes were subjected to various concentrations of miltefosine (Cayman Chemical) (0–20 µM for *L. infantum* wild type axenic amastigotes and 0–10 µM for the *Lin*PIWI null mutant).

### Plasmid Constructs and Targeting Cassettes for Gene Replacement and Transfections

The vector pSPBT1YNEOα-HA*Lin*PIWIHA was constructed as follows. The YNEOα fragment where Y is a 92 bp polypyrimidine stretch [67], NEO the neomycin phosphotransferase gene for resistance to G418, and α the intergenic region of the *L. enriettii* alpha-tubulin gene was amplified from vector pSPYNEOαLUC and inserted into NotI site of pSPBT1 [68]. The *Lin*HA-PIWI-HA was amplified from *L. infantum* genomic DNA using the primers specified in Table S1 and cloned into the EcoRV site of pSPBT1YNEOα. To generate vector pGEMαNEOα-*Lmj*PIWI-GFP, the *L. major PIWI* gene was amplified (see primers in Table S1) and cloned into the HindIII site of pGEMαNEOα-GFP vector. The *Lin*N_52_PIWI-GFP construct was made as follows. A 156 bp fragment corresponding to the first 52 amino acids of the *L. infantum* PIWI protein was amplified from *L. infantum* genomic DNA and fused with GFP (primers are described in Table S1). The fused *Lin*N_52_PIWI-GFP product was cloned into BamHI-XbaI sites of pSP72αNEOα. The purified plasmids were transfected into *L. infantum* promastigotes as described previously [68] and episomal expression of *Lin*PIWI*-*GFP and *Lin*N_52_PIWI-GFP recombinant strains were monitored by fluorescence. A targeted gene replacement strategy was used to inactivate the *L. infantum* (*Lin*) *PIWI* (LinJ.21.0470, http://tritrypdb.org) and *L. major LmjPIWI* (LmjF.21.0410, http://tritrypdb.org) genes. The 5′ and 3′ flanking regions of the *PIWI* gene (amplified from *L. infantum* and *L. major* genomic DNA independently) were fused to the hygromycin phosphotransferase (*HYG*) gene using a PCR fusion-based strategy. The 5′ flank, and 3′ flank regions were amplified from both *L. infantum* and *L. major* using primers described in Table S1. The *HYG* gene was amplified from the pSP72αHYGα *Leishmania* expression vector. The 5′-flank, *HYG* gene and 3′-flank regions were fused in a single PCR reaction using Phusion® High-Fidelity DNA Polymerase (NEB) and 5′ flank forward and 3′-flank reverse primers. The reverse primer of the 5′ flank region (5′ flank-reverse) has a tail that is complementary to the *HYG* forward primer. Similarly, the *HYG* reverse primer has a tail, which is complementary to the 3′ flank forward primer (Table S1). The resulting *HYG* cassettes corresponding to the *L. infantum* and *L. major* targeting constructs were transfected independently into both species. The two alleles of *LinPIWI* and *LmjPIWI* single copy genes were replaced by the *HYG* expression cassette following a single round of transfection by loss of heterozygocity using higher concentrations (200–600 µg/ml) of Hygromycin B (Sigma) as described previously [69].

### DNA, RNA and Protein Blots

Genomic DNA and total RNA were isolated from *L. major* and *L. infantum* strains using the DNAzol and TRIzol reagents (Invitrogen), respectively following the manufacturer’s instructions. Southern and northern blot hybridizations were performed following standard procedures [70]. Double-stranded DNA probes were radiolabeled with [α-^32^P] dCTP using random oligonucleotides and Klenow fragment DNA polymerase I [(New England Biolabs). Enrichment of small (≤200 nt) RNA fraction was carried out using the *mir*Vana™ miRNA Isolation Kit (Ambion) according the manufacturer’s procedure. The enriched small RNAs were labelled with [γ-^32^P]ATP and polynucleotide kinase (NEB) and resolved on a 15% Urea-polyacrylamide gel (National Diagnostics). The digitonin-fractionated proteins (see below) were resolved on SDS-PAGE and western blots were performed with different antibodies following standard procedures [70].

### Primer Extension Analysis

Primer extension was performed with the SuperScript™ III RT kit (Invitrogen) as described previously [49]. The forward primer corresponding to nucleotides 101–118 of the sense LSU γ rRNA (see Table S1) was used to detect antisense (as) LSU γ rRNA fragmentation. Total RNA was isolated from *L. infantum* wild type and *Lin*PIWI ^(−/−)^ mutant axenic amastigotes and used for reverse transcriptase reactions with [γ-^32^P] ATP-labeled forward primer to detect asLSU γ RNA cleavage products. Radiolabeled cDNA products were resolved on 10% Urea acrylamide gel (Sequagel, National Diagnostics) and visualized by autoradiography. A ΦX174 DNA/HinfI dephosphorylated DNA marker (Promega) was labeled with [γ-^32^P]ATP and PNK (New England Biolabs) according to the manufacturer’s recommendations.

### DNA Microarray Analysis

Probes for DNA microarray hybridizations were prepared with 10 µg of total RNA for each condition. Purified cDNA from *L. infantum* axenic amastigotes, array pre-hybridization, hybridization and washings were carried out as described previously [38]. Four biological replicates of all hybridizations were performed to account for sample heterogeneity, variation between slides and variations due to hybridization. To prevent bias by preferential label incorporation into particular sequences, Alexa 555 and Alexa 647 dyes were swapped between the two RNA preparations. The fluorescence signal intensities of the slides hybridized with *L. infantum* wild type and *LinPIWI*−/− amastigote RNA were measured using the Perkin Elmer ScanArray Express Scanner. GenePix Pro 6.0 image analysis software (Axon Instruments, Union City, California, USA) was employed to measure the fluorescence signal intensities of the array features and local background. Data correction and normalization and statistical analyses were carried out as described [38]. Only genes statistically significant with an absolute log2 ratio greater than 0.75 were considered as differentially expressed. Gene ontology annotation was analyzed using the AmiGO website [71].

### Protein Immunolocalization Studies

For immunolocalization studies of *Lin*N_52_PIWI-GFP, 10^7^ *L. infantum* promastigotes and axenic amastigotes were labeled with 20 nM MitoTracker Red (Invitrogen), a dye to stain mitochondria in live cells, in DMSO and incubated for 30 min. For *Lin*PIWI-GFP, fluorescence images were acquired through a 60×1.4 NA objective (PlanApo, Olympus) and captured at serial optical sections at 1 µm intervals by an Olympus FLUOWVIEW FV300 laser scanning confocal microscope with a 488 nm Argon-ion laser for the green channel and a 543 nm He-Ne laser for the red channel at a resolution 1024×1024 pixel. Images were produced in Fluoview software (FV300 version 5). For immunolocalization studies of the mitochondrial HSP70 protein, the parasites were fixed with 2% paraformaldehyde, permeabilized with Triton X100 (Sigma) and reacted with an anti-rabbit HSP70 antibody as a first antibody (kindly provided by Dr Osvaldo de Melo Neto, Centro de Pesquisas Aggeu Magalhães, Fiocruz, Recife, Brazil; [72]) and an anti-rabbit Alexa 594 as the second antibody. The cells were washed twice with HEPES-NaCl (500 µl) and observed under a Nikon epifluorescence microscope.

### Digitonin Fractionation

Exponentially growing *L. infantum* transfected with the *Lin*HA-PIWI-HA construct were harvested and washed twice in 1XPBS buffer and subjected to digitonin fractionation as described previously [73]. Four fractions were obtained using increasing concentrations of digitonin (20 µM, 200 µM, 1 mM and 10 mM). Proteins were precipitated with equal volume of acetone at −20°C for 1 h and re-solubilized in 1X Laemmli buffer and separated by SDS-PAGE. Western blots were performed with antibodies against the HA epitope (1∶2500), the cytoplasmic (1/1000) HSP70 and the mitochondrial HSP70 (1/1000) proteins.

## Supporting Information

Figure S1 **The N-terminal signal peptide sequence of the** ***Leishmania*** **PIWI-like protein targets the PIWI protein to the mitochondrion.** (A) Schematic representation of the *Lin*N_52_PIWI-GFP construct used for subcellular localization studies in *L. infantum* promastigotes. The *Lin*N_52_PIWI-GFP construct was made by fusing the N-terminal 52 amino acids of *Lin*PIWI (harboring the mitochondrial signal peptide sequence as predicted by SignalP 4.0, TargetP 1.1, PSORT and SOSUIsignal) to the GFP protein and then cloned into pGEM-αNEOα-GFP vector and transfected into *L. infantum* promastigotes. (B) Immunolocalization studies of *Lin*N_52_PIWI-GFP in *L. infantum* promastigotes (Pro) and amastigotes (Ama) using a Nikon epifluorescence microscope. Mitotracker (red) was used for defining the *Leishmania* single mitochondrion. The green fluorescence signal (GFP) was largely co-localized with that of the Mitotracker (red).
(TIF)

Figure S2 **Small RNA profile in** ***Leishmania*** **wild type and the PIWI^(−/−)^ null mutant.** (A) Small RNAs (<200 nt) from *L. infantum* wild type (*Lin*WT) and *Lin*PIWI−/− null mutant were isolated using the mirVanaTM Kit (Ambion), 5′-labelled by polynucleotide kinase and loaded on denaturing 15% SDS-PAGE gel. More RNA fragments are seen in amastigotes (A) (this could also be due to degradation products) than in promastigotes (P) but there is no significant accumulation or decrease of small RNA species in the *Lin*PIWI^(−/−)^ mutant in comparison to wild type cells. The marker (M) corresponds to an end-labeled 20 nt DNA oligonucleotide. (B) Small RNAs isolated as in A and enriched by 15–45% sucrose gradient analysis to look for small RNAs associated or not with ribosomes. No significant differences in small RNA species between wild type and the *Lin*PIWI^(−/−)^ mutant were observed.
(TIF)

Figure S3 **RNA editing is fully operational in the** ***L. infantum*** **PIWI^(−/−)^ null mutant.** The cDNAs were generated from total RNA of wild type and *Lin*PIWI^(−/−)^ axenic amastigotes by random hexamers. The cDNAs were used for PCR amplification for cytochrome C oxidase subunit II (*COXII*) (A, upper panel) using specific primers and sequenced. The edited sequence of *COXII* in which four uridines were added is indicated by red arrows (A, lower panel). The PCR fragment amplified from unedited mitochondrial DNA sequence was shown as control for this experiment (B, upper panel) cDNA PCR amplification for the cytochrome b (*CYB*) gene using specific primers. (B, lower panel) Nucleotide sequence of the *CYB* gene with the edited uracils underlined in red. There is no difference in the editing pattern of *COXII* (A) and *CYB* (B) transcripts between *L. infantum* wild type and *Lin*PIWI^(−/−)^ mutant. The experiment was repeated twice with similar results. The PCR framgment amplified from unedited mitochondrial DNA sequence was shown as control.
(TIF)

Figure S4 **Kinetoplastid DNA pattern between the** ***L. infantum*** **wild type and** ***Lin*** **PIWI^(−/−)^ null mutant.** Ethidium bromide-stained gel of circular kinetoplastid DNA (kDNA) from both *L. infantum* wild type (*Lin*PIWI^(+/+)^) and *Lin*PIWI^(−/−)^ null mutant. The circular kinetoplastid DNA was isolated by plasmid mini-preparation columns (Qiagen), digested with HindIII and EcoRI enzymes and run on a 1% agarose gel. No differences were observed in kDNA between the *L. infantum* wild type and *Lin*PIWI^(−/−)^ null mutant.
(TIF)

Figure S5 **Expression vectors to rescue the** ***Lin*** **PIWI^(−/−)^ null mutant.** Schematic representation of the *Lin*PIWI gene fused or not at either extremity with known epitope tag sequences (e.g. HA, MYC and GST) and cloned into the BamHI and XbaI sites of vector pSP72αNEOα (see Materials and Methods). These constructs were transfected into the *L. infantum Lin*PIWI^(−/−)^strain to rescue the null mutant phenotype.
(TIF)

Table S1 **Primers used in this study.**
(DOC)
