# Supplementary figures and images for: Novel Features of a PIWI-Like Protein Homolog in the Parasitic Protozoan Leishmania
Source: PLoS One. 2012 Dec 21;7(12):e52612. doi: 10.1371/journal.pone.0052612 (PMC3528672; doi:10.1371/journal.pone.0052612)

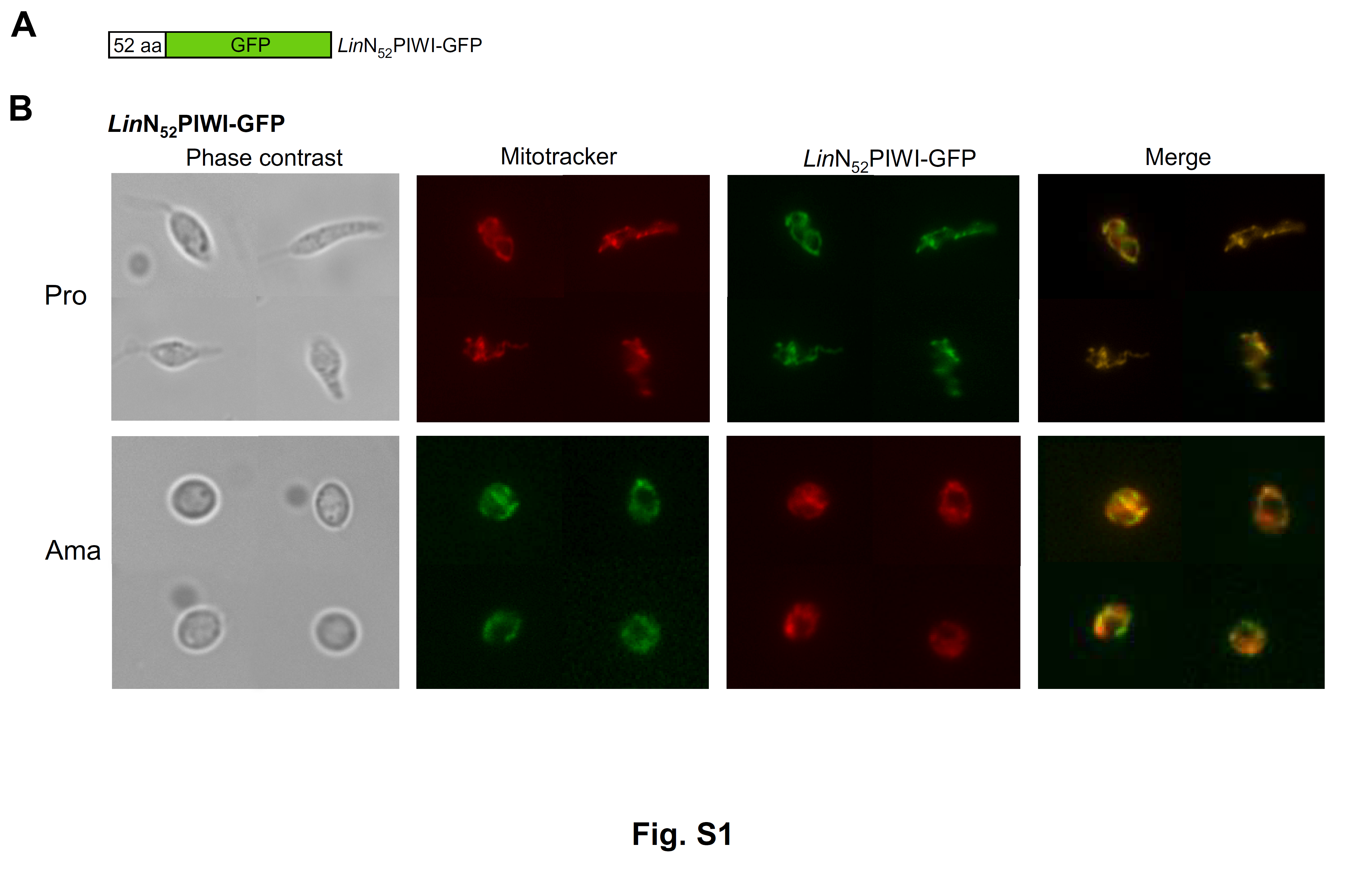

Supplement: Figure S1 — The N-terminal signal peptide sequence of the Leishmania PIWI-like protein targets the PIWI protein to the mitochondrion. (A) Schematic representation of the LinN52PIWI-GFP construct used for subcellular localization studies in L. infantum promastigotes. The LinN52PIWI-GFP construct was made by fusing the N-terminal 52 amino acids of LinPIWI (harboring the mitochondrial signal peptide sequence as predicted by SignalP 4.0, TargetP 1.1, PSORT and SOSUIsignal) to the GFP protein and then cloned into pGEM-αNEOα-GFP vector and transfected into L. infantum promastigotes. (B) Immunolocalization studies of LinN52PIWI-GFP in L. infantum promastigotes (Pro) and amastigotes (Ama) using a Nikon epifluorescence microscope. Mitotracker (red) was used for defining the Leishmania single mitochondrion. The green fluorescence signal (GFP) was largely co-localized with that of the Mitotracker (red). (TIF) [file pone.0052612.s001.tif]

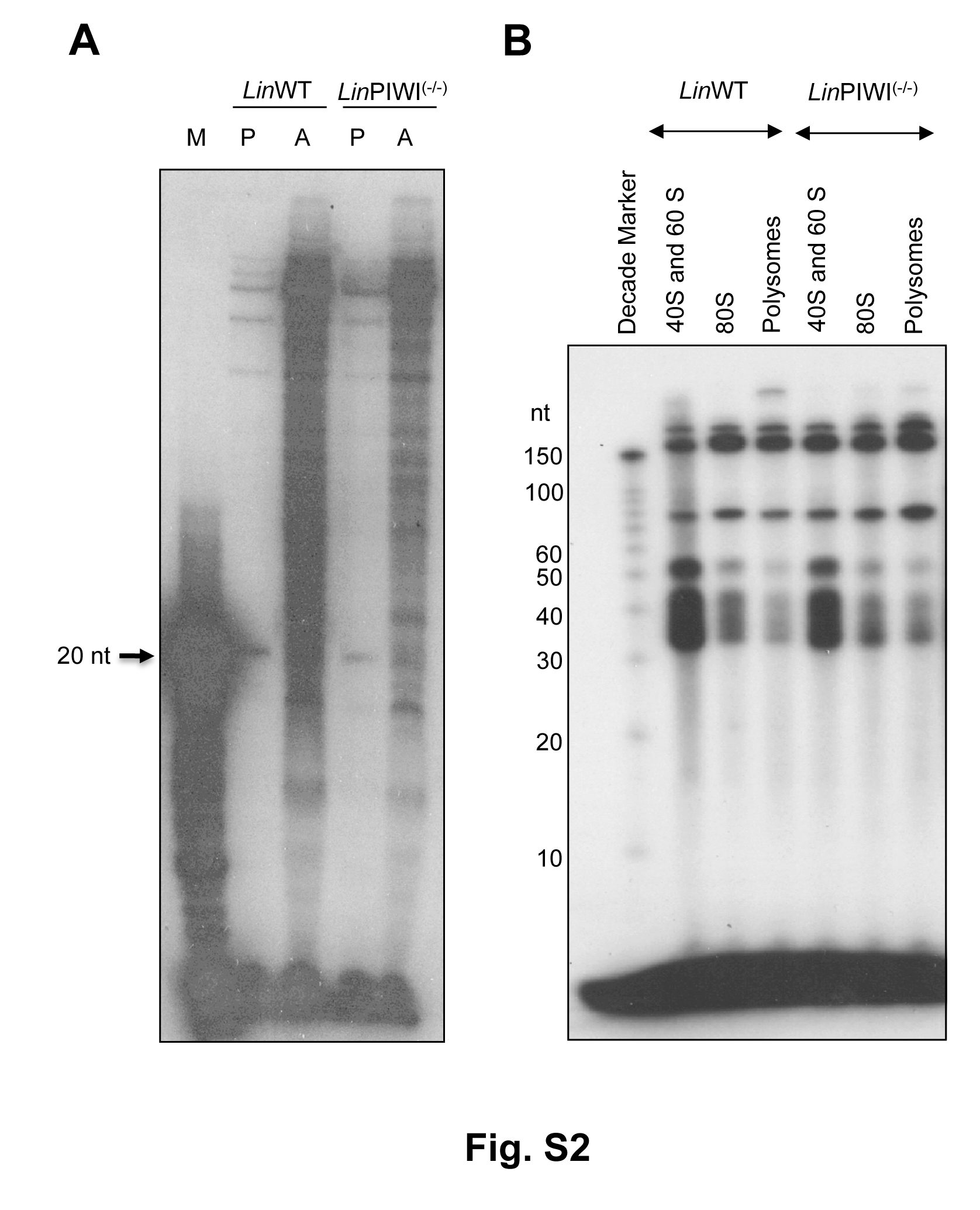

Supplement: Figure S2 — Small RNA profile in Leishmania wild type and the PIWI(−/−) null mutant. (A) Small RNAs (<200 nt) from L. infantum wild type (LinWT) and LinPIWI−/− null mutant were isolated using the mirVanaTM Kit (Ambion), 5′-labelled by polynucleotide kinase and loaded on denaturing 15% SDS-PAGE gel. More RNA fragments are seen in amastigotes (A) (this could also be due to degradation products) than in promastigotes (P) but there is no significant accumulation or decrease of small RNA species in the LinPIWI(−/−) mutant in comparison to wild type cells. The marker (M) corresponds to an end-labeled 20 nt DNA oligonucleotide. (B) Small RNAs isolated as in A and enriched by 15–45% sucrose gradient analysis to look for small RNAs associated or not with ribosomes. No significant differences in small RNA species between wild type and the LinPIWI(−/−) mutant were observed. (TIF) [file pone.0052612.s002.tif]

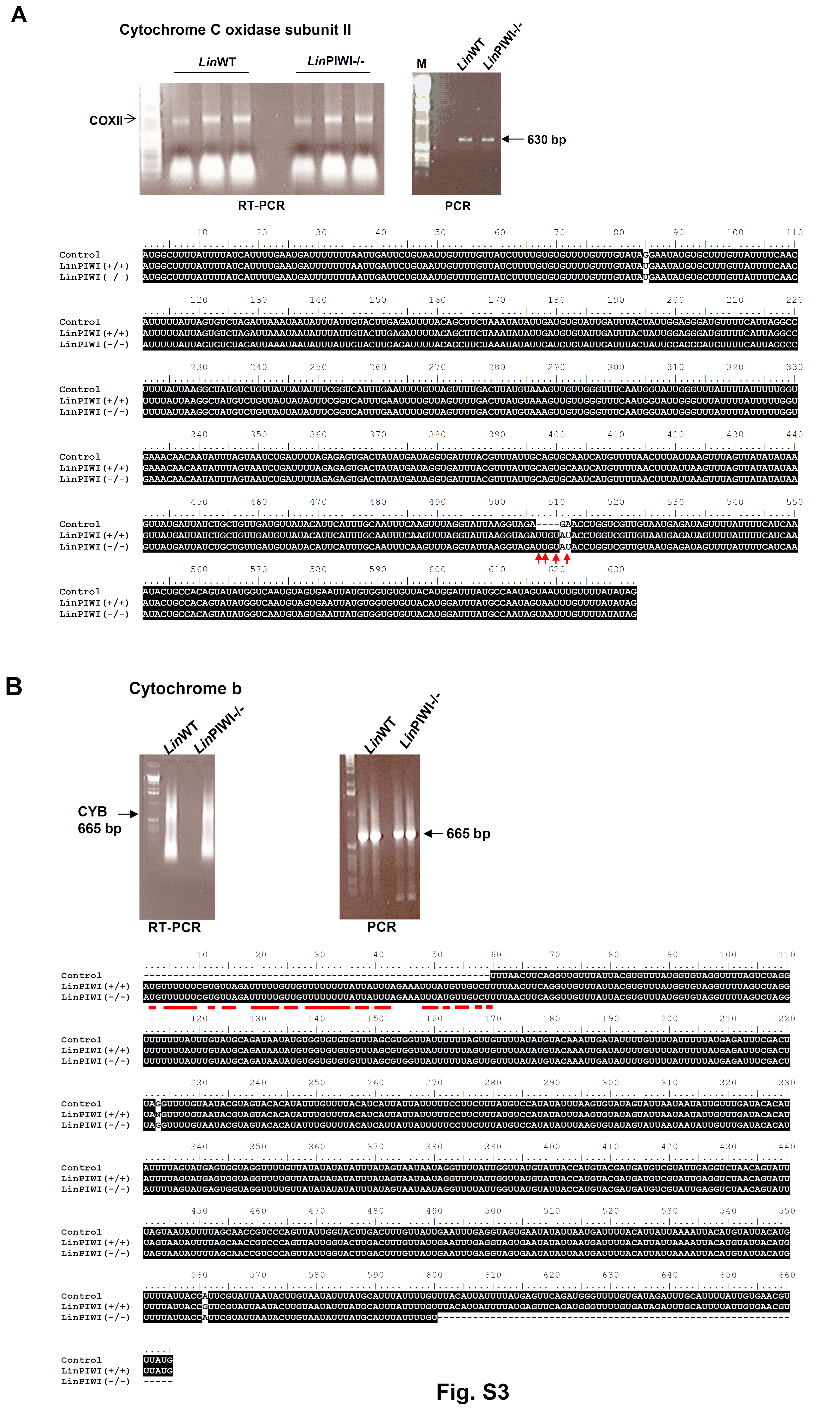

Supplement: Figure S3 — RNA editing is fully operational in the L. infantum PIWI(−/−) null mutant. The cDNAs were generated from total RNA of wild type and LinPIWI(−/−) axenic amastigotes by random hexamers. The cDNAs were used for PCR amplification for cytochrome C oxidase subunit II (COXII) (A, upper panel) using specific primers and sequenced. The edited sequence of COXII in which four uridines were added is indicated by red arrows (A, lower panel). The PCR fragment amplified from unedited mitochondrial DNA sequence was shown as control for this experiment (B, upper panel) cDNA PCR amplification for the cytochrome b (CYB) gene using specific primers. (B, lower panel) Nucleotide sequence of the CYB gene with the edited uracils underlined in red. There is no difference in the editing pattern of COXII (A) and CYB (B) transcripts between L. infantum wild type and LinPIWI(−/−) mutant. The experiment was repeated twice with similar results. The PCR framgment amplified from unedited mitochondrial DNA sequence was shown as control. (TIF) [file pone.0052612.s003.tif]

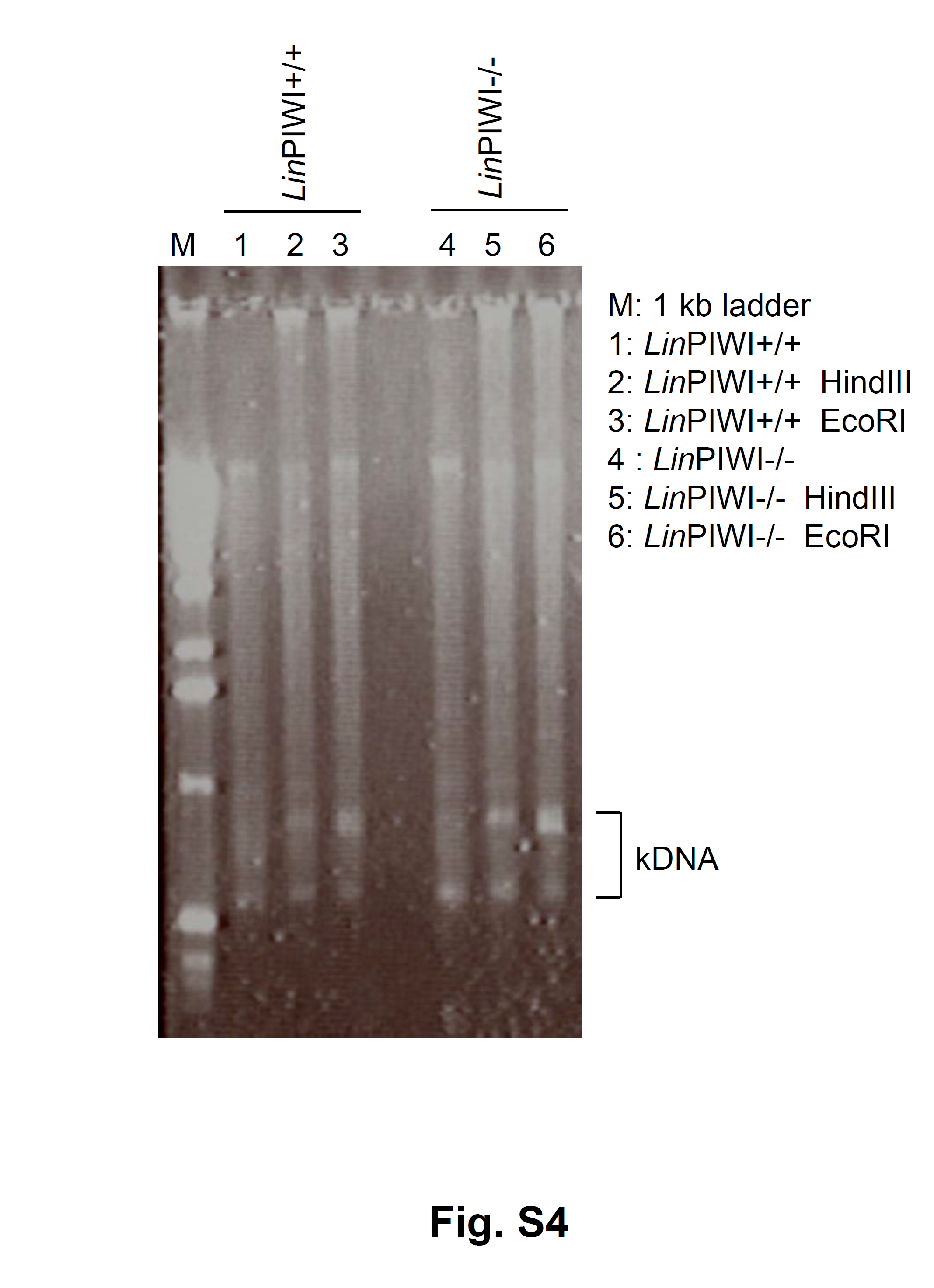

Supplement: Figure S4 — Kinetoplastid DNA pattern between the L. infantum wild type and Lin PIWI(−/−) null mutant. Ethidium bromide-stained gel of circular kinetoplastid DNA (kDNA) from both L. infantum wild type (LinPIWI(+/+)) and LinPIWI(−/−) null mutant. The circular kinetoplastid DNA was isolated by plasmid mini-preparation columns (Qiagen), digested with HindIII and EcoRI enzymes and run on a 1% agarose gel. No differences were observed in kDNA between the L. infantum wild type and LinPIWI(−/−) null mutant. (TIF) [file pone.0052612.s004.tif]

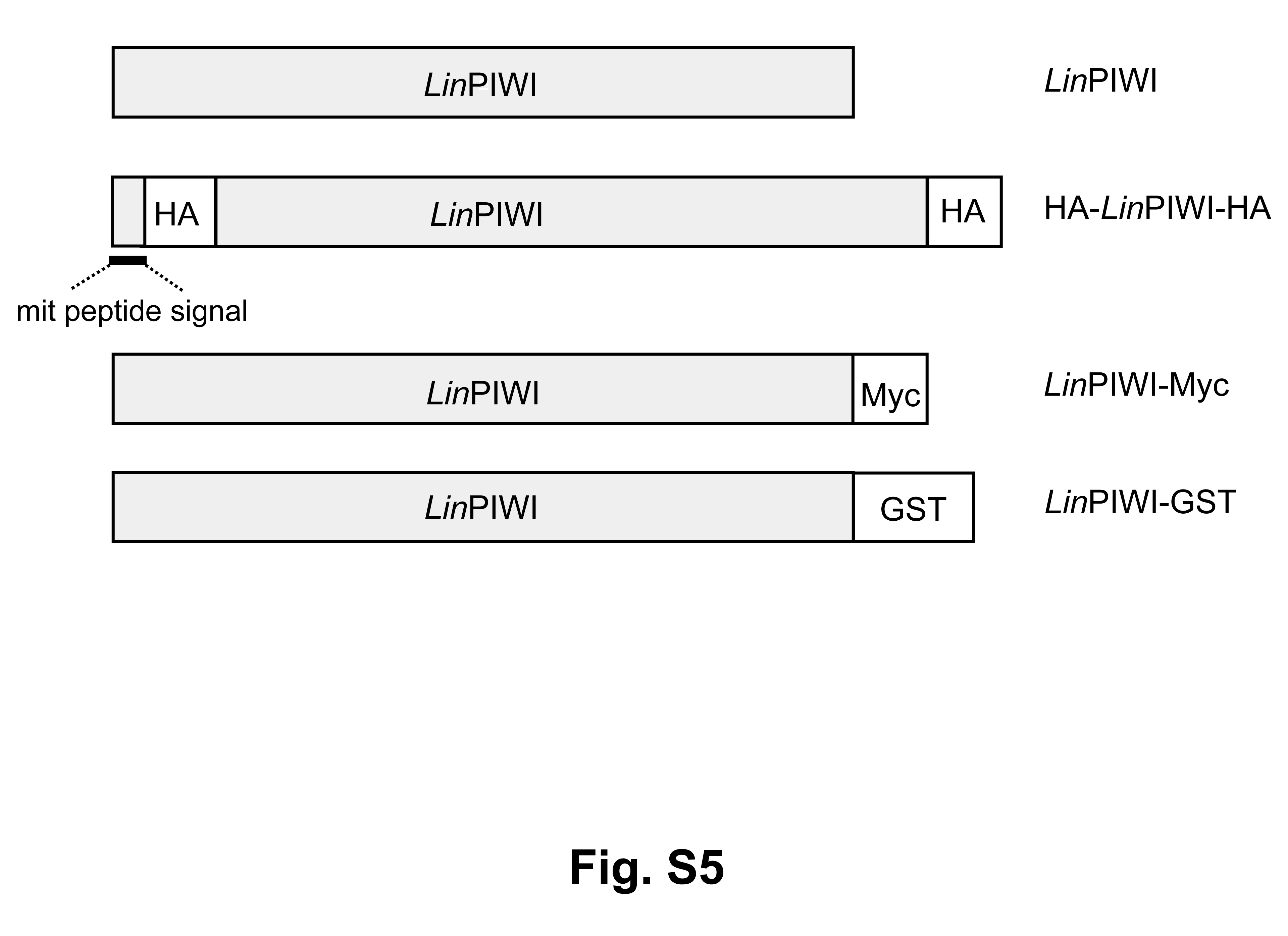

Supplement: Figure S5 — Expression vectors to rescue the Lin PIWI(−/−) null mutant. Schematic representation of the LinPIWI gene fused or not at either extremity with known epitope tag sequences (e.g. HA, MYC and GST) and cloned into the BamHI and XbaI sites of vector pSP72αNEOα (see Materials and Methods). These constructs were transfected into the L. infantum LinPIWI(−/−)strain to rescue the null mutant phenotype. (TIF) [file pone.0052612.s005.tif]
