# Supplementary material for: Novel Features of a PIWI-Like Protein Homolog in the Parasitic Protozoan Leishmania
Source: PLoS One. 2012 Dec 21;7(12):e52612. doi: 10.1371/journal.pone.0052612 (PMC3528672; doi:10.1371/journal.pone.0052612)
Supplement: Table S1 — Primers used in this study. (DOC) [file pone.0052612.s006.doc]

**Table S1.** Primers used in this study.

| **PIWI overexpression constructs** |  |  |
| --- | --- | --- |
| *Lmj*PIWI primers  *Lin*PIWI forward  *Lin*PIWI reverse | 5’- CGGGATCCATGTGGTCCCTACTCCGCCCAAG-3’  5’- GCTCTAGACTAGAGAAACCACAAGCGGTTGCAC-3’ |  |
| *Lmj*PIWI-GFP primers  *Lmj*PIWI forward  *Lmj*PIWI reverse | 5’- CCCAAGCTTATGTGGTCCCTACTCCGCCCAAG-3’  5’- CCCAAGCTTGAGAAACCACAGGCGGTTGCAC-3’ |  |
| *Lin*N_52_PIWI-GFP primers  *Lin*N_52_PIWI (156 bp region) forward  *Lin*N_52_PIWI (152 bp region) reverse  *GFP* forward  *GFP* reverse | 5’-CGGGATCCATGTGGTCCCTACTCCGCCCAAG-3’  5’-GAACAGCTCCTCGCCCTTGCTCACCCCGTCGGAGTAGCCGTGGTAG-3’  5’-CTACCACGGCTACTCCGACGGGGTGAGCAAGGGCGAGGAGCTGTTC-3’  5’-GCTCTAGATTACTTGTACAGCTCGTCCATGCCGAGA-3’ |  |
| HA-*Lin*PIWI-HA primers  HA-*Lin*PIWI-HA forward  HA-*Lin*PIWI-HA reverse | 5’-**CGGGATCCATG**TACCCTTACGACGTGCCAGACTACGCT**TGGTCCCTACTCCGCCCAAG**-3’  5’**GCTCTAGACTA**AGCGTAGTCTGGCACGTCGTAAGGGTA**GAGAAACCACAAGCGGTTGCACAG**3’ |  |
| *Lin*PIWI-Myc primers  *Lin*PIWI-Myc forward  *Lin*PIWI-Myc reverse | 5’- CGGGATCCATGTGGTCCCTACTCCGCCCAAG-3’  5’GCTCTAGACTAGAGGTCTTCCTCGCTGATTAGCTTCTGCTCGAGAAACCACAAGCGGTTGCACAG3’ |  |
| *Lin*PIWI-GST  *Lin*PIWI-GST forward  *Lin*PIWI-GST reverse  GST forward  GST forward | 5’-CGGGATCCATGTGGTCCCTACTCCGCCCAAG-3’  5’-GCTCTAGAGAGAAACCACAAGCGGTTGCAC-3’  5’-GCTCTAGA ATGTCCCCTATACTAGGTTATTG-3’  5’-CCGGTCGACTCAATCCGATTTTGGAGGATG-3’ |  |
| **Probes for northern blot hybridization** |  |  |
| *Linj.19.0030*  *H2B* forward  *H2B* reverse | 5`-TCGCACCGCAAGCCTAAG-3’  5`- TTCGTGCCCTCAGCCATG-3’ |  |
| *LinJ.10.0920*  *H3* forward  *H3* reverse | 5`-ATGTCCCGCACCAAGGAG-3’  5`-GTCCTTCGGCTGGATCGT-3’ |  |
| *LinJ.21.0020*  *H4* forward  *H4* reverse | 5`-CGCTCCGCTGATGCCAAG--3’  5`-TTACGCGTAGCCGTAGAGGATG-3’ |  |
| **PIWI gene targeting constructs** |  |  |
| Primers for amplifying the 5’flank region of the *L. infantum* *Lin*PIWI gene    5’flank forward  5’flank reverse | 5’-CGGGATCCagttcacagaaacgcaacgag-3’  5’-GGTGAGTTCAGGCTTTTTCATccttcagatttctgcgggggtg-3’ |  |
| Primers for hygromycin gene amplification  *HYG* forward  *HYG* reverse | 5’-ATGAAAAAGCCTGAACTCACC-3’  - 5’-GTGACACCGCCATGTGCCGCTATTCCTTTGCCCTCGGACGAG-3’ |  |
| Primers for amplifying the 3’ flank region of the *Lin*PIWI gene  3’flank forward  3’flank reverse | 5’- cggcacatggcggtgtcac -3’  5’- CCCAAGCTTacacgcaaggggcgacgtg -3’ |  |
| HYG targeting cassette for *L. major*  Primers for amplifying the 5’flank region of the *Lmj*PIWI gene  5’flank forward  5’flank reverse | 5’-Actctgcattggcggaaaggag-3’  5’-GGTGAGTTCAGGCTTTTTCATgcttcagatctctgcgggggtg -3’ |  |
| Primers for *HYG* gene amplification  *HYG* forward  *HYG* reverse | 5’-ATGAAAAAGCCTGAACTCACC -3’  5’-gtgagaccgccacgtaccGCTATTCCTTTGCCCTCGGACGAG-3’ |  |
| Primers for amplifying the 3’ flank region of the *Lmj*PIWI gene  3’flank forward  3’flank reverse | 5’- Cggtacgtggcggtctcac -3’  5’-CCCACACACACACACACGCAAG -3’ |  |
| **Primers used for investigating for RNA editing**  COXII forward  COXII reverse  CYTB forward  CYTB reverse | 5’- ATGGCTTTTATTTTATCA-3’  5’- ctatataaaacaaattactattg-3’  5’-ATGTTTTTTCGTGTTAGATTTTTG-3’  5’- CATAAACGTTCACAATAAAATG-3’ |  |
| **Primer extension analysis**  *Leishmania infantum* LSU γ 101-118 forward | 5’-cctttttacttctcgcgt-3’ | 5’-cctttttacttctcgcgt-3’ |
